# 

**Published:** 1883-03-20

**Authors:** 


### TABLE OF CONTENTS.

ORIGINAL AND SELECTED ARTICLES.

Reflex-Neuroses and Nose Diseases: Contribution to Nasal Surgery, by Dr. William Hauck, Privat-docent in the Freiburg University, 81. Lupus Exedens, with the History of a case Successfully Treated by the use of the Solar Ray Cautery, by O. V. Thayer, M. D., 86. Premature Delivery for the Prevention of Blindness, by Edward G. Loring, M. D., of N. Y., 89. Treatment of Acute Rheumatism, 93. Look out foi Your Soft Catheters, by Hiram Nance, M. D., 95. Sulphate of Zinc as a Specific in Scarlet Fever, by Dr. W. D. Hoyt, of Rome, Ga, 96. Capsicum Enemata in Opium Poisoning, by James G. Kiernan, M. D., Chicago, Illinois, 98. Health of Criminal Women; Good-bye to the Doctor, 99.

ABSTRACTS AND GLEANINGS.

Koch's Theory of Tuberculosis, 100. Meniere's Disease; Electricity, 103. Ophthalmic Aphorisms, 104. Tracheotomy, 105. New Treatment for Purulent Conjunctivitis; Coffee as an Antidote to Alcoholism, 106. Infantile Convulsions, 107. Vertigo de Meniere; Carbolic Poison; Therapeutics of Chloral Hydrate, 108. Alcohol Inhalations in Croupal Diphtheria, 109. Jaborandi in Jaundice; A New Method of Embalming; Collecting Bills; A New Narcotic; Specific for Sore Throat. 110.

SCIENTIFIC ITEMS.

Some Large Lenses; The average; Lightning Rods, 111. A Curious Experiment; The Climate of Palestine, 112.

PRACTICAL NOTES AND FORMULÆ.

Antiseptic Cologne; Cough Mixtures; The Metric System, 113. Diarrhœa Pills; Comparative Temperatures; Facts Worth Knowing, 114.

EDITORIALS AND MISCELLANEOUS.

Southern Medical College Commencement, 115. Editorial Notices, 118. Book Notices, 119. Receipted; Special Notices, 120.

